# Sexual Violence in University Sports Teams: Female Student-Athletes’ Experiences in Three Quebec Higher Education Institutions

**DOI:** 10.1177/10778012261425950

**Published:** 2026-04-10

**Authors:** Maïa Savard, David Bezeau, Félix Berrigan

**Affiliations:** 1 Faculty of Physical Activity Sciences, Department of Kinanthropology, 7321 University of Sherbrooke, Sherbrooke, Québec, Canada

**Keywords:** sexual violence, female student-athletes, university sports teams, Quebec, Canada

## Abstract

This study provides a descriptive overview of sexual violence (SV) experienced by female student-athletes (*n* = 56) from three Quebec universities, examining the frequencies, forms, and contexts of these incidents. Findings show that over one-third of participants (43%, *n* = 24) reported at least one SV incident perpetrated by someone linked to their university sports team. Most incidents were perpetrated by men, primarily fellow student-athletes, and occurred most frequently during social activities. These findings highlight that SV remains a current issue despite existing prevention efforts and provide information that could support the development of more effective prevention strategies and educational initiatives in university sports.

## Introduction

In recent years, public disclosures and media coverage of sexual violence (SV) incidents on university campuses and within sports teams in Canada, including the province of Quebec, have drawn attention to the persistence and severity of the problem (Institut national de santé publique du Québec, 2024). In response, both federal and provincial authorities have implemented a range of policies and initiatives to prevent and address SV. For example, the Government of Quebec, one of Canada's ten provinces with its own legislative framework, passed in 2017 the Act to prevent and fight SV in higher education institutions, requiring all universities to implement comprehensive prevention and response policies (RLRQ, c. P-22.1). Complementary initiatives, such as Je dénonce (Gouvernement du Québec, 2026) and Canadian Safe Sport Program (Sport Integrity Canada, 2026), were created to centralize the reporting of abuse and harassment within sports federations. Despite these measures, SV remains prevalent on university campuses and in sports environments. Since student-athletes navigate both university and sport settings, they may represent a particularly vulnerable population, underscoring the importance of understanding the specific forms, frequencies, and contexts of SV they may experience to guide effective prevention and intervention efforts.

### Research Objectives

While similar issues of SV on university campuses and in sports teams exist across Canada and internationally, Quebec's distinctive policy and institutional framework provides a concrete example of how governments and institutions have responded to SV, as well as a relevant backdrop for documenting the forms and contexts of SV experienced by student-athletes. However, to our knowledge, no prior study in Quebec has specifically examined SV experienced by student-athletes when the perpetrators are individuals affiliated with university sports teams. Therefore, this study seeks to fill that gap by providing a descriptive overview of SV perpetrated specifically by athletes, coaches, or members of the technical, administrative, medical, or strength and conditioning staff, whether these incidents take place inside or outside sport settings, on campus, or in virtual environments. By doing so, the study aims to contribute to a better understanding of student-athletes’ experiences and the context in which SV occurs.

Specifically, this article addresses the following research questions: (1) What are the frequencies and forms of SV experienced by female student-athletes from three Quebec universities, and (2) In what contexts do these incidents occur? The focus on female student-athletes in this article is justified both methodologically and substantively. Methodologically, the study draws on a cross-sectional survey completed by 78 varsity student-athletes. Although 21 male student-athletes completed the survey, their small number precluded meaningful analysis. Therefore, to align with the article's objectives and avoid statistically unreliable comparisons, the present findings focus on the female subsample (*n* = 56). Analyses are limited to descriptive statistics to provide a clear overview of the data without making inferential claims.

Substantively, women are consistently identified in the literature as a higher-risk group for experiencing SV in university and sports contexts (Bergeron et al., 2016; DeStefano et al., 2024; Vertommen et al., 2016). By centering on female student-athletes, the study provides detailed insights into the prevalence, forms, and contexts of SV in a particularly vulnerable population, which is essential for guiding future research and informing educational and prevention strategies aimed at fostering safer and more inclusive university sports environments. Having established the research questions, we now turn to a review of the literature to contextualize the prevalence, perpetrators, and contexts of SV within university and sports settings.

### Literature Review

#### Sexual Violence at University

The prevalence of SV on university campuses has been increasingly documented over the past decades. Early empirical work by DeKeseredy and Schwartz (1998) provided one of the first national surveys documenting SV on Canadian university campuses, showing that a substantial proportion of women experienced sexual assault, unwanted sexual contact, or harassment, and highlighting the role of peer dynamics in shaping these experiences. These patterns remain relevant today, as recent official government data from Statistics Canada (2019) indicate that 44.6% of women and 32.2% of men experienced at least one unwanted sexualized behavior in a post-secondary education context (Burczycka, 2020). These national figures are complemented by targeted institutional studies. For example, Jeffrey et al. (2022) conducted a survey at one Canadian university and found that 23.2% of women, 9.6% of men, and 16.7% of nonbinary students experienced at least one incident of SV in the past 12 months, with women reporting significantly greater trauma and academic impacts than men. Similarly, an online survey conducted across universities in Ontario, a Canadian province, found that 63.2% of students reported experiencing sexual harassment and 23% reported at least one nonconsensual sexual experience during their university studies, with prevalence rates of sexual assault, sexual harassment, and stalking for women and gender-diverse respondents higher than the overall university sector rates (Ontario Ministry of Training, Colleges and Universities, 2019). Data from the Quebec-based Study on Sexuality, Security and Interactions on a University Campus: What Students, Professors and Employees are Saying [ESSIMU] also revealed disturbing rates of SV among students (36.2%), with women (40.6%) significantly more likely than men (26.4%) to report incidents of SV perpetrated by another individual affiliated with the university (Bergeron et al., 2016, 2019). International data provide additional context. A national French survey found that since they arrived in higher education, 24% of women and 9% of men have experienced at least one form of sexual assault or rape (or attempt), often repeatedly (Bègue-Shankland, 2024). In the United States, a systematic review revealed SV prevalence in universities ranging from 6% to 44.2% among female students, covering experiences of unwanted sexual touching, rape, and attempted rape (Fedina et al., 2018). Collectively, these findings consistently indicate that women report higher rates of campus SV than men, both in Canada and internationally, highlighting the persistent gendered nature of SV in post-secondary settings (Banyard et al., 2004; Steele et al., 2024; Walsh et al., 2010).

Beyond prevalence, existing research has also shed light on the profiles and characteristics of SV perpetrators. Evidence indicates that perpetrators are primarily students targeting their peers (Bergeron et al., 2016; Burczycka, 2020; Hill & Silva, 2005). Cases involving perpetrators in positions of authority are reported in these studies, such as professors targeting students, but they appear to be less frequent. Several studies also show that SV in post-secondary settings is most frequently perpetrated by men, regardless of the victim's gender (Bergeron et al., 2016; Bègue-Shankland, 2024; Burczycka, 2020; Hill & Silva, 2005).

Some research has also examined the contexts in which such violence occurs. In the study by Bergeron et al. (2016), social or party-related activities were identified as the most common contexts for SV incidents perpetrated by individuals affiliated with the university. These findings echo those of Bègue-Shankland (2024), who reports that more than half of sexual and gender-based violence in university settings involves alcohol consumption by either survivors or perpetrators, a pattern that the frequent association between festive social activities and alcohol use may explain. More precisely, alcohol was present in 62% of sexual assault attempts, 56% of sexual assaults, and 43% of rapes in this study. Additionally, certain university subcultures, such as competitive athletic programs and fraternities, have been identified as higher-risk environments for SV (Martin, 2016; McCray et al., 2023; Moylan & Javorka, 2020).

#### Sexual Violence in Sport

Some studies suggest that sports participation may offer a form of protection against SV throughout athletes’ lives (Fasting et al., 2003; Parent et al., 2016), particularly among varsity athletes (Fasting et al., 2008). Fasting et al.'s (2003) “Sport Protection Hypothesis” explains this phenomenon by suggesting that athletes develop strength, self-confidence, and physical adaptability through their sports experiences, which may serve as a defense against SV.

On the other hand, research has documented how the competitive and social dynamics of sports can heighten risks of exploitation, maltreatment, and violence against athletes, including SV (Fasting et al., 2002; Leahy et al., 2002; Mountjoy et al., 2016; Ohlert et al., 2019; Vertommen et al., 2016; Willson et al., 2022). Several studies have provided prevalence estimates that illustrate the scope of the problem. For example, Willson et al. (2022) found that 20.5% of Canadian national team athletes reported experiencing at least one form of SV during their careers. Parent et al. (2016) documented SV among adolescent athletes in organized sports, with 8.8% reporting victimization, a figure that likely underestimates the true prevalence given the study's exclusion of peer-perpetrated SV and noncontact unwanted sexual behaviors. Together, these findings suggest that SV occurs across competitive levels, though prevalence rates vary depending on methodological scope and definitions used. Moreover, Ohlert et al. (2019) showed that athletes who had experienced SV reported lower well-being and a higher risk of depressive symptoms, underscoring that the psychological impacts of SV in sport mirror those observed among SV victims more broadly.

While some studies report that female athletes experience higher rates of SV (Vertommen et al., 2016; Ohlert et al., 2018; Willson et al., 2022), others found no significant differences between genders, with male and female athletes reporting similar rates (Fasting et al., 2008; Parent et al., 2016; Parent & Vaillancourt-Morel, 2021). Differences in study methodologies, sampling strategies, and the specific definitions of SV employed may explain those variations. Literature also sheds light on the nature of the perpetrators, indicating that SV in sports settings is more frequently perpetrated by athlete peers than by authority figures, although some cases in smaller proportions also involve coaches and other authority figures (Alexander et al., 2011; Willson et al., 2022).

#### Sexual Violence in University Sports Teams

While research on SV in university sports settings has largely focused on student-athletes as perpetrators (Bonar et al., 2022; McCray, 2015), limited attention has been given to SV experienced by them. In addition, existing studies on this topic offer inconclusive findings, partly due to their heterogeneous methodologies and definition of SV (McCray et al., 2023). Some studies have found no statistically significant difference in SV risk between student-athletes and non-athletes in university settings (Parent et al., 2016; McCray et al., 2023). On the other hand, several studies report non-negligible rates of victimization among varsity athletes, suggesting that this population may face distinct vulnerabilities (Adhia et al., 2023; DeStefano et al., 2024; Parent et al., 2016). For instance, Adhia et al. (2023) found that 29% of American undergraduate student-athletes experienced SV since enrolling in university, with a higher prevalence of SV among student-athletes on women's teams (36%) compared to men's teams (13%). DeStefano et al. (2024) reported an even higher rate, with 44% of American student-athletes disclosing at least one incident of SV in their lifetime, including unwanted touching, forced sexual acts, nonconsensual sex, and sexual harassment. However, neither study specifically examines SV occurring within the sports context or involving perpetrators affiliated with a university sports team.

Similarly, in Quebec, a study by Parent et al. (2022) examined whether participation in varsity sports is associated with an increased or decreased risk of experiencing SV on university campuses. Based on a subsample from the ESSIMU survey (Bergeron et al., 2016), the authors reported that 42% of varsity athletes had experienced at least one incident of SV since starting university. Among these cases, a small proportion identified a coach as the perpetrator (7%), while the vast majority (90%) reported another student. However, because the survey was not specifically designed for athletes, it remains unclear whether these student perpetrators were also part of a university sports team. Results from this study also showed that identifying as female was associated with a higher likelihood of experiencing various forms of campus SV within the past year, regardless of varsity athlete status. This gendered vulnerability is further supported by DeStefano et al. (2024), who reported that female university athletes face significantly higher rates of SV compared to their male counterparts.

Several factors specific to the university sports environment may contribute to this heightened vulnerability of varsity student-athletes. Studies have shown that female and male student-athletes are more likely to adhere to rape myths (i.e., beliefs that shift blame onto victims and excuse perpetrators) than non-athlete students, which can hinder their ability to recognize or report experiences of SV (McMahon, 2019; Young et al., 2017). This increased risk may be further exacerbated by higher alcohol consumption and athletes’ tendency to isolate themselves from other social groups, primarily interacting with teammates and fellow athletes, including male student-athletes who can be at a higher risk for sexually aggressive behavior (Bonar et al., 2022; Brown et al., 2013). Additionally, some studies suggest that the risk of SV among student-athletes may be partly attributed to athletic cultures that promote or normalize problematic attitudes, such as hypermasculinity, which have been associated with an increased risk of SV perpetration (Murnen & Kohlman, 2007; Young et al., 2017). Within this context, the pressure to conform and integrate into the team can create facilitating conditions for SV. On the other hand, some studies are suggesting that the heightened vulnerability of student-athletes may be more closely linked to other known risk factors, rather than their status as athletes or their participation in sports per se (Parent et al., 2022; McCray et al., 2023). For example, Parent et al. (2022) found that the higher incidence of SV reported among varsity athletes was largely explained by factors unrelated to sport, such as being younger, more likely to be undergraduates, Indigenous, or international students.

### Sexual Violence Definition

In the present study, the term “sexual violence” is used inclusively to cover a broad spectrum of forms and manifestations of SV. It is therefore not limited solely to acts defined as sexual assaults under the Canadian Criminal Code. More precisely, SV is defined as any form of violence expressed through sexually motivated behaviors or those targeting sexuality, including sexual assault. This definition also includes other types of misconduct, such as gestures, words, behaviors, or attitudes with unwanted sexual connotations, including those targeting sexual and gender diversity. These behaviors may be expressed directly or indirectly, including through technological means (RLRQ, 2017, c.P-22.1).

This definition of SV is grounded in a feminist framework, which frames such violence beyond legal categories, through a gendered and continuum-based lens (Bergeron et al., 2016). The notion of a continuum makes it possible to encompass a wide range of manifestations and forms of SV, most of which primarily target women, without attempting to rank them by severity (Bergeron et al., 2016; Rousseau et al., 2020). Developed by Kelly (1987), the continuum approach has been widely adopted and updated in the scientific literature to address contemporary issues, including those related to gender diversity, sexual orientation, and digital environments (Bergeron et al., 2016). This perspective is essential, as it brings attention to forms that are socially trivialized, such as sexually suggestive comments or jokes, which occur far more frequently than criminal acts or forms of violence perceived as explicitly aggressive, such as physical assault (Savoie et al., 2019).

## Methods

### Participants

Following ethics review board approval, student-athletes from three participating universities were recruited to answer a confidential online survey via online solicitation, posters, and email invitations between November 2023 and March 2024. Only students who were currently members of one or more university sports teams were eligible to participate, which was confirmed by the survey's first question. The three universities collectively have approximately 1,200 varsity student-athletes based on available estimates, though the exact number who received the survey invitation is unknown, preventing calculation of a precise response rate. A total of 129 participants responded. After excluding 51 incomplete surveys, 78 complete surveys remained for analysis. The present findings focus on the female subsample (*n* = 56) to align with the article's objectives and avoid statistically unreliable comparisons. Frequencies for male student-athletes (*n* = 21) are presented in tables for informational purposes only. The sociodemographic profile of the 78 participants is presented in Table 1.

**Table 1.** Sample Sociodemographic Characteristics (*n* = 78).

	*n* (%)
Affiliated university (*n* = 78)	
Sherbrooke University	42 (54%)
Laval University	7 (9%)
University of Quebec in Montreal	29 (37%)
Age (*n* = 78)	
Under 18	1 (1%)
19–24	67 (86%)
25–30	9 (12%)
31+	1 (1%)
Student status (*n* = 78)	
Undergraduate student	61 (78%)
Master's student	14 (18%)
PhD student	3 (4%)
Gender identity (*n* = 78)	
Female	56 (72%)
Male	21 (27%)
Nonbinary/Trans	1 (1%)
Sexual identity (*n* = 83)^a^	
Heterosexual	61 (74%)
Sexual minorities	19 (23%)
Uncertain/questioning	3 (4%)
International status	7 (9%)
Visible minority status	8 (10%)
Disability status	1 (1%)

a The cumulative rates do not equal the number of respondents in the sample (*n* > 78) since individuals may identify with more than one sexual orientation.

### Measures

#### Sexual Violence in University Sports Teams

SV was measured using an adaptation of the ESSIMU survey (Bergeron et al., 2016), which is a French adaptation of the Sexual Experiences Questionnaire (Fitzgerald et al., 1999; SEQ-DoD). Section 1 of the survey aims to collect the participants’ sociodemographic characteristics and athletic experiences. Section 2 seeks to document the frequencies of SV incidents committed by a person affiliated with a university sports team (athletes, coaches, and members of the technical, administrative, medical, or strength and conditioning staff), occurring either within or outside the sports context. Measures of section 2 included a total of 21 items and three subscales: (1) sexual harassment (i.e., sexual verbal and nonverbal behaviors that do not aim at sexual cooperation but manifest as insulting, hostile, and degrading attitudes), (2) unwanted sexual contact (i.e., offensive, unwanted, and nonreciprocal verbal and nonverbal sexual behaviors, including sexual assault, attempted rape, and rape), and (3) sexual coercion (i.e., abuse of power, blackmail, pressure, or threats to consent to sexual activities in exchange for future considerations related to sports or other matters). The sexual harassment subscale consists of eight items (e.g., “Did someone make insulting or hurtful comments that were sexual in nature?”). The unwanted sexual contact subscale has seven items (e.g., “Did someone try to have sexual relations with you against your will?”). The sexual coercion subscale has six items (e.g., “Did someone make you suffer negative consequences because you refused to engage in sexual activities with them?”). Participants indicated on a five-point scale how many times a person affiliated with a university sports team committed each of the 21 items toward them since joining a university sports team (never, once, two or three times, four or five times, and more than five times) and whether each occurred at least once over the last 12 months (yes or no). The subscale scores were dichotomized (0 = no victimization and 1 = at least one event).

#### Contexts of Sexual Violence

Participants who reported SV were asked additional questions regarding the context of these experiences. For each reported item, they answered multiple-choice questions to indicate their status at the time of the incident (undergraduate student, master's student, or PhD student), the gender and status of the perpetrators (e.g., “another athlete”, “a coaching staff member”, “a medical staff member”, etc.) and the physical context in which the SV occurred, such as “In a sports context (training, practice, tournament, competition, etc.)” or “During a party or another social event”. Since participants could report multiple SV incidents, they were able to provide multiple responses to these follow-up questions. As a result, our data do not provide a per-incident contextual picture, which limits the ability to determine whether particular context factors co-occur within the same incident or across different incidents. While this approach reduced participant burden, it introduces a methodological limitation that should be considered when interpreting the contextual findings.

### Analysis

Descriptive analyses were conducted using Excel, following the simple percentage and combined percentage methods proposed by Fitzgerald et al. (1999) and used in Bergeron et al. (2016). Given the small and imbalanced sample sizes, no inferential statistical comparisons were performed, and all frequencies and percentages are presented for descriptive purposes only.

### Ethics

This study was approved by the University of Sherbrooke Research Ethics Committee in Education and Social Sciences, by the Université of Québec in Montreal Research Ethics Committee, and by the Laval University Research Ethics Committee in Psychology and Educational Sciences. All participants provided written informed consent before participating.

### Limitations

It is important to acknowledge certain limitations of this study that may influence the interpretation of the findings. First, the sample's representativeness is limited, as it is small and was drawn from only three universities in Quebec, preventing generalization to all university sports teams in the province. Second, the sample composition is not proportional in terms of several sociodemographic characteristics, which also limits the representativeness of the results. For example, there is a higher proportion of respondents from the University of Sherbrooke and fewer from Laval University. Additionally, there is an imbalance in gender representation, with substantially fewer male respondents than female respondents, which precluded meaningful gender comparisons. Lastly, the exclusion of 51 surveys due to missing responses suggests a potential motivational bias, possibly related to the sensitivity or length of the survey.

## Findings

### Frequencies and Forms of Sexual Violence Experienced by Female Student-Athletes in Three Quebec Universities

Table 2 presents frequencies of SV incidents among student-athletes. Over one-third of female student-athletes (43%, *n* = 24) indicated having experienced at least one incident of SV perpetrated by an individual affiliated with a university sports team, since joining a university sports team. In the 12 months prior to the study, nearly all female victims (*n* = 23/24) reported experiencing at least one incident of SV.

**Table 2.** Sexual Violence Frequencies Experienced Among Student-Athletes (n = 77).

	Female Student-Athletes (*n* = 56)	Male Student-Athletes (*n* = 21)
Sexual Harassment	23 (41%)	6 (29%)
Unwanted Sexual Behavior	11 (20%)	3 (14%)
Sexual Coercion	2 (3%)	0 (0%)
At least one incident of SV	24 (43%)	6 (29%)
SV Victimization—in the last 12 months		
Sexual Harassment	22 (39%)	6 (29%)
Unwanted Sexual Behavior	9 (16%)	2 (10%)
Sexual Coercion	2 (3%)	0 (0%)
At least one incident of SV	23 (41%)	6 (29%)

*Note.* Frequencies for male student-athletes are presented for informational purposes only and were not included in further analyses due to the small sample size and the article's focus on female student-athletes.

Sexual harassment was the most frequently reported form of SV. The data reveal that 41% (*n* = 23) of female student-athletes experienced at least one incident of sexual harassment since joining a university sports team, such as “*Repeatedly told you sexual stories or jokes that you found offensive,”* “*Attempted to start a conversation about sex with you, even if you were uncomfortable (e.g., tried to discuss your sex life with you),”* or “*Stared at you or undressed you with their eyes in a way that made you feel uncomfortable.”* Regarding forms typically considered more severe, 5% (*n* = 4) of student-athletes who reported experiencing unwanted sexual behaviors indicated having been subjected to forced sexual intercourse by an individual affiliated with a university sports team, all of whom were women. Additionally, only two participants reported incidents of sexual coercion and both were women.

### Female Student-Athletes’ Contexts of Sexual Harassment, Unwanted Sexual Behaviors, and Sexual Coercion

Descriptive analyses were conducted using the sample of women who reported at least one form of SV against them (*n* = 24). Questions regarding the context of each SV incident documented the victim's status at the time, the perpetrator's identity, and the location where the incident occurred. Table 3 shows that for each respective form of SV, most victims were undergraduate students at the time of the incidents. Specifically, 87% (*n* = 20) of the 23 women who reported sexual harassment were undergraduates, as were 73% (*n* = 8) of the 11 victims of unwanted sexual behaviors, and 100% (*n* = 2) of the victims of sexual coercion. Among the three PhD students in the sample, none reported experiencing SV.

**Table 3.** Victim and Perpetrator Characteristics Among Female Student-Athletes (*n* = 24), by Form.

	Sexual Harassment Victims (*n* = 23)^a^	Unwanted Sexual Behavior Victims (*n* = 11)	Sexual Coercion Victims (*n* = 2)
Victim status			
Undergraduate student	20 (87%)	8 (73%)	2 (100%)
Master's student	4 (17%)	3 (27%)	0 (0%)
PhD student	0 (0%)	0 (0%)	0 (0%)
Perpetrator status			
Athletes	19 (82%)	7 (64%)	1 (50%)
Coaching staff member	3 (13%)	3 (27%)	0 (0%)
Technical staff member	2 (9%)	1 (9%)	0 (0%)
Strength and conditioning staff member	1 (4%)	0 (0%)	0 (0%)
Unidentified status	0 (0%)	0 (0%)	1 (50%)
Medical staff member	0 (0%)	0 (0%)	0 (0%)
Perpetrator gender			
Female	7 (30%)	2 (18%)	1 (50%)
Male	18 (78%)	9 (82%)	1 (50%)

a The cumulative percentage does not always equal 100% for the “Sexual Harassment” column, as one woman reported being a victim under two different statuses (first as an undergraduate student and later as a graduate student) and two women reported being assaulted by both females and males.

The distributions of perpetrator characteristics varied across SV forms. Most victims of sexual harassment (82%, *n* = 19) and of unwanted sexual behaviors (64%, *n* = 7) reported being assaulted by a fellow athlete. Descriptive analyses also revealed that 13% (*n* = 3) of sexual harassment victims and 27% (*n* = 3) of victims of unwanted sexual behaviors reported incidents involving a head coach or assistant coach. Regarding the two victims of sexual coercion, one woman identified the perpetrator as another athlete, while the second chose not to disclose the perpetrator's status. For all three forms of SV, no perpetrator was identified as a member of the medical staff (physiotherapist, massage therapist, doctor, athletic therapist, intern, etc.).

Regarding perpetrator gender, most of the sexual harassment (78%, *n* = 18) and unwanted sexual behaviors (82%, *n* = 9) victims reported being assaulted at least once by a man. Females were identified as the perpetrator by 30% (*n* = 7) of sexual harassment victims and 18% (*n* = 2) of victims of unwanted sexual behaviors. Among the two victims of sexual coercion, one reported being assaulted by a man and the other by a woman.

Each participant may have experienced and reported more than one SV incident in the survey. Therefore, the percentages shown in Table 4 refer to the number of reported incidents per form and in total, rather than the number of victims. The data reveal that SV incidents across all forms (*n* = 114) most frequently occur in the context of parties or social activities (39%), followed closely by sports settings (37%). Nearly half (49%) reported incidents of unwanted sexual behaviors took place during parties or social activities. In contrast, sexual harassment incidents were more commonly reported in sports settings (42%), followed closely by parties or social activities contexts (36%). Finally, more than half (60%) of the reported sexual coercion incidents occurred in sports settings. All female student-athletes who reported at least one form of SV against them (*n* = 24) reported at least one incident in sports settings and at least one incident during parties or social activities.

**Table 4.** Contexts in Which Sexual Violence Incidents Occurred (*n* = 114) Among Female Student-Athletes.

	Sexual Harassment Incidents (*n* = 69)	Unwanted Sexual Behavior Incidents (*n* = 35)	Sexual Coercion Incidents (*n* = 10)	SV Incidents (Total) (*n* = 114)
Parties/social activities	25 (36%)	**17** (**49%)**	3 (30%)	**45** (**39%)**
Sport context	**29** (**42%)**	7 (20%)	6 (60%)	**42** (**37%)**
Online	5 (7%)	4 (11%)	0 (0%)	9 (8%)
Sport initiation	3 (4%)	2 (6%)	1 (10%)	6 (5%)
Student involvement	4 (6%)	2 (6%)	0 (0%)	6 (5%)
Work	3 (4%)	1 (3%)	0 (0%)	4 (4%)
Teaching context	0 (0%)	2 (6%)	0 (0%)	2 (2%)
Faculty or departmental initiation	0 (0%)	0 (0%)	0 (0%)	0 (0%)

## Discussion

### Female Student-Athletes’ Forms of Sexual Violence

Regarding the first objective, two main findings will be discussed: (1) sexual harassment was the most common form of SV experienced, and (2) despite their overall lower reporting rates, incidents of rape and sexual coercion were disclosed only by female student-athletes in the sample.

Of the female student-athletes reporting at least one SV incident (43%, *n* = 24), the majority experienced sexual harassment, making it the most frequently reported form (41%, *n* = 23). This highlights that a substantial portion of reported SV incidents involved behaviors often trivialized, such as unwanted sexual comments, gestures, or persistent advances. Similar patterns have been observed in Canadian and Quebec studies, where sexual harassment is the most commonly reported form of SV among university students, including student-athletes. For instance, the ESSIMU survey shows that 33% of students and 38% of student-athletes have experienced sexual harassment, with female students reporting higher rates of victimization than their male counterparts (Bergeron et al., 2019; Parent et al., 2022). National studies, such as Burczycka (2020) and Willson et al. (2022), further document the prevalence of unwanted sexual jokes, comments, and attention, particularly among elite athletes who frequently encounter sexist or sexual remarks and intrusive gazes. Jeffrey et al. (2022) also found that nonpenetrative sexual contact was the most common nonconsensual sexual experience for all university students in their sample. These findings align with the SV continuum framework, which suggests that behaviors associated with this type of violence tend to occur more frequently than criminalized behaviors or those socially regarded as severe, particularly among women (Kelly, 1987). This may be due to persistent prejudices and harmful beliefs in the university community, where behaviors such as sexual comments or jokes are often viewed as trivial, minor actions or unjustly interpreted as SV (Bergeron et al., 2016; Savoie et al., 2019). Yet, studies have found that these experiences can have significant consequences for well-being and sense of safety in sport or in university (Bergeron et al., 2016; Johansson et al., 2024). For example, a recent Swedish study found that female students exposed to sexual harassment, such as unwelcome sexual comments, gestures, or attention, reported significantly higher symptom levels of depression and anxiety, illustrating the mental health impact of these seemingly “minor” behaviors (Johansson et al., 2024). By documenting these dynamics, our study highlights the need for prevention efforts that reflect the lived experiences of varsity student-athletes, addressing not only severe or criminalized forms of SV but also the normalized, “everyday” behaviors that can profoundly impact their well-being and sense of safety.

Consistent with the SV continuum and existing literature, forms typically and socially considered more severe (such as coercive sex, attempted rape, or rape) were reported less often than other SV forms in the present study (Fedina et al., 2018; Steele et al., 2024). Despite being reported less frequently, these forms of SV remain a significant concern, particularly given the focused and limited scope of our data. Specifically, among student-athletes who reported experiencing unwanted sexual behaviors, 5% (*n* = 4) indicated having been subjected to forced sexual intercourse by an individual affiliated with a university sports team. These findings echo those of Fasting et al. (2008), who reported a slightly higher lifetime prevalence (7%) of forced sexual intercourse among American collegiate student-athletes. However, unlike our study, their data did not specifically focus on incidents committed by individuals affiliated with university sports teams. Additionally, the fact that all four victims of rape and both victims of sexual coercion in our sample were women raises important questions about the gendered dynamics of SV within the context of university sports. This finding aligns with broader trends in SV research, which consistently show that women, particularly in male-dominated spaces such as sports, are more affected by forms of violence that are often considered more severe or that may carry more serious physical, psychological, and social consequences, such as rape (Burczycka, 2020; Brackenridge et al., 2008; Jeffrey et al., 2022). For instance, Jeffrey et al. (2022) reported that female students were disproportionately affected by nonpenetrative sexual contact, attempted rape, and rape, with 7.1% of women indicating they had experienced rape compared to only 2.0% of men. Similarly, the national French survey by Bègue-Shankland (2024) confirms this, revealing that female students are 2.7 to 4.5 times more likely to be victims than male students of sexual assault attempts, sexual assault, attempted rape, or rape. A national study by Statistics Canada also found that women are more often the target of potentially criminal behaviors than men, suggesting that significant disparities exist between the SV experiences of women and those of men in post-secondary environments (Burczycka, 2020). In sport settings, Brackenridge et al. (2008) found that 17% of female athlete victims reported experiencing rape, whereas none of the male victims did, while Rintaugu et al. (2014) identified that 28% of university female athletes reported attempted rape. These results underscore the importance of analyzing SV in university sports through a theoretical feminist lens that accounts for the gendered inequalities and structural dynamics that shape women's experiences in these spaces (Bergeron et al., 2016). Although our sample size did not allow for formal statistical comparison between genders, the fact that only women reported rape or sexual coercion in our descriptive data further illustrates the need to account for these dynamics when designing prevention strategies.

### Female Student-Athletes’ Contexts of Sexual Violence

Regarding the second objective, the following three main findings will be discussed: (1) most individuals who perpetrated SV against female student-athletes are fellow student-athletes; (2) men were most frequently identified as the perpetrators; and (3) SV incidents across all forms most frequently occur in the context of parties or social activities, followed closely by sports settings.

The present study found that most female student-athletes who were victims of sexual harassment (82%, *n* = 19) and unwanted sexual behaviors (64%, *n* = 7) indicated a fellow student-athlete from university sports teams as the perpetrator. This result is unsurprising, given that student-athletes typically spend most of their time with teammates during training, competitions, classes, and social activities rather than with any other individuals affiliated with university sports. The strong peer dynamics within teams may also increase the likelihood of intra-group interactions, both positive and negative (Elendu & Umeakuka, 2011). This result aligns with existing research showing that acts of SV in sports, including university sports teams, are more frequently committed by athletes against peers than by individuals in positions of authority (Alexander et al., 2011; Elendu & Umeakuka, 2011; Vertommen et al., 2016 ; Willson et al., 2022). Similar patterns have also been observed in studies on SV in university settings more broadly, where most of the reported incidents involve students assaulting other students (Bergeron et al., 2016; Burczycka, 2020; Hill & Silva, 2005; Parent et al., 2022). However, these studies do not allow us to determine whether the student perpetrators were themselves members of university sports teams. The present study helps fill this gap in the scientific literature by addressing a limitation identified by Parent et al. (2022): “While participants identified the gender of the perpetrator, we did not ask their perpetrator's athlete status. […]. Those characteristics could help better understand the real risk posed by male varsity athletes towards other athletes for sexual violence in university settings. […] Further research could deepen our understanding of this question.” (p. 9).

Regarding the gender of the perpetrators, most female victims reported that at least one incident of sexual harassment (78%, *n* = 18) and unwanted sexual behaviors (82%, *n* = 9) involved a male perpetrator. These results are consistent with prior research indicating that SV in university settings is predominantly perpetrated by men (Bergeron et al., 2016; Burczycka, 2020; Hill & Silva, 2005; Jeffrey et al., 2022). For example, the ESSIMU study reported that 91% of students and 92% of varsity athletes who had experienced at least one form of SV identified a male perpetrator (Bergeron et al., 2019; Parent et al., 2016). Similarly, the French study by Bègue-Shankland (2024) found comparable rates, with male perpetrators identified in 90% to 95% of cases, regardless of the type of SV experienced by students. Lastly, Jeffrey et al. (2022) reported that the majority of victims of attempted (84.2%) and completed (92.1%) rape, most of whom were women, identified men as the perpetrators. Such high rates highlight the importance of understanding SV within the broader context of men's violence against women. Nevertheless, although male perpetrators were predominant, a notable proportion of victims in this study also identified female perpetrators: 30% (*n* = 7) in cases of sexual harassment and 18% (*n* = 2) in cases of unwanted sexual behaviors. This is also supported by Parent et al. (2016), where 30% of varsity athletes reported that the perpetrator's gender was female. Despite these findings, research on SV in sports involving female perpetrators remains limited, highlighting a significant gap in the existing literature.

Lastly, our data allowed us to identify where the incidents of SV perpetrated by someone affiliated with a university sports team had occurred. Parties or social activities (39%) were the most common environment reported by female student-athletes across all forms of SV. The frequent occurrence of SV in party or social settings aligns with broader research on campus SV, which identifies these contexts as common sites for such incidents (Bergeron et al., 2016; Burczycka, 2020; Parent et al., 2022; Walsh et al., 2010), as well as a risk factor for SV among university students (Moylan & Javorka, 2020). Burczycka (2020) suggests that the significant proportion of SV in party and social settings may be linked to alcohol and drug consumption. This is supported by Bègue-Shankland (2024), who reports that over half of sexual and gender-based violence incidents in university settings involve alcohol consumption. The sports setting (37%) emerged as the second most frequently reported context for SV incidents, which is not surprising given that both victims and perpetrators in this study had to be affiliated with a university sports team. Furthermore, the significant amount of time student-athletes dedicate each week to sport-related activities may increase the likelihood of SV occurring in this environment. On the other hand, Parent et al. (2022) found that only 13% of SV incidents reported by student-athletes in the ESSIMU sample occurred in a sports setting. This lower rate may be explained by methodological factors, as the ESSIMU survey was not specifically designed to investigate SV within university sports teams. Additionally, the small sample size in the present study may have contributed to the higher frequencies of SV incidents reported in sports settings. Overall, these findings underscore the importance of considering all settings in which student-athletes may experience SV perpetrated by members of university sports teams, regardless of the event's location.

### Strengths

Despite the methodological limitations, the current study has several strengths. Most importantly, to our knowledge, this study is the first to provide insight into the characteristics of SV experienced by Quebec varsity student-athletes, specifically when the perpetrator is affiliated with a university sports team. While the study focuses on Quebec, its findings are relevant beyond this province, as similar challenges related to SV exist across Canada and internationally. Secondly, by considering a broad range of SV forms, not only those typically perceived as severe, this study also contributes to a more nuanced understanding of the risks and dynamics specific to student-athletes. Finally, using quantitative data from a subsample of female student-athletes, the study offers valuable information on the status of both victims and perpetrators, as well as the contexts in which these incidents occurred. Such an analysis is particularly important given that women from our sample reported SV incidents more frequently than men, suggesting potential gendered patterns that could help guide future prevention efforts.

## Conclusion

Overall, our results contribute to the literature by documenting high frequencies of SV incidents among student-athletes in three Quebec universities, with most victims having experienced these incidents within the past 12 months prior to the study. These findings underscore that SV remains a pressing issue affecting a substantial portion of student-athletes in our sample, despite ongoing prevention efforts. This highlights the need to examine the effectiveness of current strategies and explore additional evidence-informed approaches. For instance, our results could support mandatory bystander intervention training for athletes and coaching staff, an approach shown to increase participants’ confidence and willingness to intervene in risky situations (Hébert et al., 2018).

Our data further demonstrate that SV occurs along a continuum, from frequent sexual comments and jokes to acts considered more severe, such as sexual coercion or forced sexual intercourse, emphasizing the importance of a broad and inclusive conceptualization of SV, consistent with Fitzgerald et al.'s (1999) framework. Consequently, prevention initiatives should ideally equip athletes to recognize and respond to all forms of SV, including behaviors that may be perceived as minor but are widespread and impactful. If current programs do not yet fully achieve this, our findings suggest that strengthening such components could contribute to evidence-informed policies that foster safer and more inclusive university sports environments while reflecting the lived realities of student-athletes.

Additionally, the high frequencies of victimization observed among female student-athletes, including forms with potentially serious consequences such as forced sexual intercourse and sexual coercion, suggest a potentially gendered pattern and point to the particular challenges that women may face in university sports. These findings reinforce the need to design preventive measures and interventions tailored to individuals at higher risk, as recommended by the Quebec Government's Act to prevent and fight sexual violence in higher education institutions (RLRQ, 2017, c. P-22.1), which calls for targeted prevention strategies and support for vulnerable student populations.

While this study provides valuable insights into female student-athletes’ experiences of SV in Quebec and can inform prevention programs, future research with larger and more balanced samples is needed to enable analytic comparisons and better assess potential gendered patterns. Larger samples would also allow for a more representative understanding of SV among other at-risk subgroups, such as sexual and gender minority or international student-athletes. Furthermore, individual interviews could provide qualitative depth to support the design of targeted and effective preventive measures, while mixed-methods approaches would capture both experiences and consequences of SV.

In conclusion, the incidents of SV reported in this study reinforce the urgency of addressing and preventing SV not only within Quebec's university sports teams, but also across varsity programs in other Canadian provinces, where similar dynamics are likely present.
